# Supplementary figures and images for: Influence of isoflurane on the diastolic pressure-flow relationship and critical occlusion pressure during arterial CABG surgery: a randomized controlled trial
Source: PeerJ. 2016 Jan 26;4:e1619. doi: 10.7717/peerj.1619 (PMC4783760; doi:10.7717/peerj.1619)

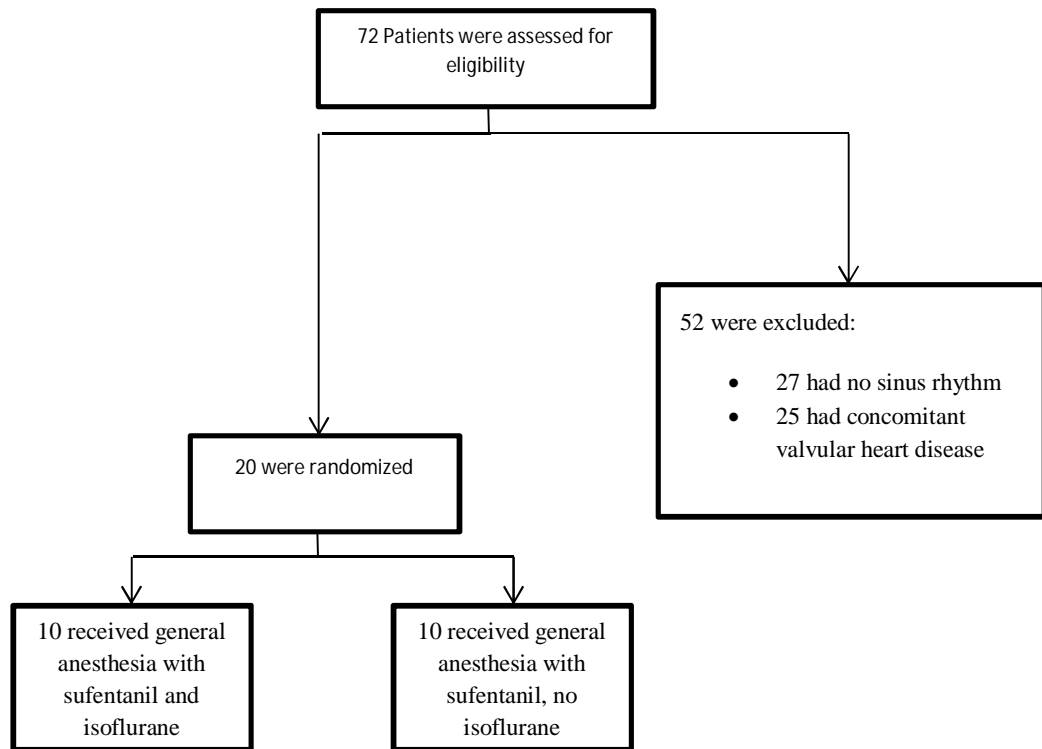

Supplement: Supplemental Information 6 — Flow diagram. [file peerj-04-1619-s006.pdf]
